# Aging Experiences Around the World: Local Findings and Global Insights From Population Surveys of Aging

**DOI:** 10.1093/geroni/igaa057.1841

**Published:** 2020-12-16

**Authors:** Jennifer Ailshire, Jinkook Lee

## Abstract

International comparisons of the aging experience offer a unique opportunity to understand how much the aging process varies according to differences in social environments. Despite the potential knowledge gained from global comparisons of health and aging, conducting cross-national comparisons can be challenging. The HRS-family of surveys, which have been harmonized within the Gateway to Global Aging, provides remarkable opportunities for cross-national comparative analysis. The papers in this session use harmonized data to compare the aging experience across different social dimensions in multiple countries from around the world, including examinations of: 1) the role of social engagement among married couples in cognitive function in Mexico and the United States; 2) the importance of work histories and macro-economic policies on later life health in England and Europe; 3) gender differences in the receipt of informal care in the U.S., Korea, and China; 4) the association between sensory impairment and disability-free life expectancy in England and the U.S.; and 5) end-of-life care arrangements and health care utilization in the context of different health systems across multiple countries.

